# Retraction: Glycation Accelerates Fibrillization of the Amyloidogenic W7FW14F Apomyoglobin

**DOI:** 10.1371/journal.pone.0218284

**Published:** 2019-06-06

**Authors:** The PLOS ONE Editors

After publication of this article [1], questions were raised about results reported in Figures 2A and 5:

- In Figure 2A, there are similarities between the lower bands shown in lane 1 of the left panel and lane 1 of the right panel, and between the upper half of lane 4 in the left and right panels. There are also abrupt changes in the background in the left panel after lane 1 at the bottom and lane 3 at the top, and in the right panel near the lower bands in lanes 1, 2, and 4.
- There are similarities between results shown in Figure 5A and 5B, suggesting that they show different fields of the same sample.

The authors clarified that an error was made in assembling Figure 5 such that the wrong image was presented in Figure 5A. They provided raw image files supporting the results in Figure 5, including an updated image of W7FW14F apomyoglobin in the absence of D-ribose at the initial timepoint.

The original western blot images supporting Figure 2A are no longer available. The authors provided replication data for this experiment but have not been able to clarify the concerns noted above. The underlying data supporting other results in this article are available upon request from the corresponding author.

In light of the unresolved concerns about Figure 2A, the *PLOS ONE* Editors retract this article.

RM did not respond. CI, GI, IS did not agree with retraction.
